# Comparative evaluation of anopheline sampling methods in three localities in Indonesia

**DOI:** 10.1186/s12936-017-2161-9

**Published:** 2018-01-08

**Authors:** Brandyce St. Laurent, Supratman Sukowati, Timothy A. Burton, David Bretz, Mulyadi Zio, Syah Firman, Sumardi, Heru Sudibyo, Amalia Safitri, Suwito, Puji B. Asih, Sully Kosasih, Shinta, William A. Hawley, Thomas R. Burkot, Frank H. Collins, Din Syafruddin, Neil F. Lobo

**Affiliations:** 1 Eck Institute for Global Health, University of Notre Dame, Notre Dame, IN USA; 2 National Institute of Health Research and Development, Jakarta, Indonesia; 3 Eijkman Institute for Molecular Biology, Jakarta, Indonesia; 4 Centers for Disease Control and Prevention, Atlanta, GA USA; 5 Unicef, Jakarta, Indonesia; 6 National Institutes of Health, Bethesda, MD USA; 7 Queensland Tropical Health Alliance, James Cook University, Australian Institute of Tropical Health and Medicine, Cairns, Australia

**Keywords:** *Anopheles*, Sampling, Vector ecology, Indonesia, Malaria

## Abstract

**Background:**

The effectiveness of vector control efforts can vary based on the interventions used and local mosquito behaviour and adaptability. In many settings, biting patterns of *Anopheles* mosquitoes can shift in response to interventions targeting indoor-biting mosquitoes, often resulting in higher proportions of mosquitoes feeding outside or at times when people are not protected. These behaviourally resistant mosquitoes have been shown to sustain residual malaria transmission and limit control efforts. Therefore, it is important to accurately sample mosquitoes to understand their behaviour.

**Methods:**

A variety of traps were evaluated in three geographically diverse sites in malaria-endemic Indonesia to investigate local mosquito feeding behaviour and determine effective traps for surveillance.

**Results:**

Eight traps were evaluated in three sites: Canti village, Lampung, Kaliharjo village, Purworejo, and Saketa village, Halmahera, Indonesia, including the gold standard human landing collection (HLC) and a variety of traps targeting host-seeking and resting mosquitoes both indoors and outdoors. Trapping, using indoor and outdoor HLC, the Ifakara tent trap C, goat and human-occupied tents, resting pots and boxes, and CDC miniature light traps was conducted for 16 nights in two sites and 8 nights in a third site, using a Latin square design. Trap efficacy varied by site, with outdoor HLC yielding the highest catch rates in Canti and Kaliharjo and a goat-baited tent trap proving most effective in Saketa. In Canti village, anthropophilic *Anopheles sundaicus* were caught indoors and outdoors using HLCs, peaking in the early morning. In Kaliharjo, a variety of mosquitoes were caught, mostly outdoors throughout the night. HLC was ineffective in Saketa, the only site where a goat-baited tent trap was tested. This trap was effective in catching zoophilic vectors outdoors before midnight.

**Conclusions:**

Different trapping methods were suitable for different species, likely reflecting differences in behaviour among species. The three villages, each located on a different island in the Indonesian archipelago, contained mosquito populations with unique behaviours. These data suggest that the effectiveness of specific vector monitoring and control measures may vary by location.

**Electronic supplementary material:**

The online version of this article (10.1186/s12936-017-2161-9) contains supplementary material, which is available to authorized users.

## Background

Between the introduction of the UN Millennium Development Goals in 2000 and 2015, incidence of malaria is estimated to have decreased by 37%. In much of Africa, this reduction is attributed to the roll-out of vector control using insecticide-treated nets (ITNs) or indoor residual spray (IRS), although the effect of these interventions in the rest of the world is less understood [1, 2]. Recent studies have shown a large diversity of vector species in endemic areas, a number of which may exhibit behaviour not targeted by existing control measures [3–5]. In many settings, biting patterns of *Anopheles* mosquitoes are shifting and insecticide resistance alleles are becoming widespread, presumably in adaptation to deployed vector control measures [6–12]. On a local level, understanding the species and behavioural diversity of resident anophelines is crucial for developing and targeting vector interventions [13].

To adequately survey local vector species, efficient sampling methods that capture relevant (i.e., human biting) vectors are needed. All sampling methods have their respective biases as they target different aspects of mosquito behavioural patterns, and some are only useful in particular environments [14]. The choice of sampling method is largely influenced by local species-specific behaviour and the entomological endpoint of interest (e.g., indoor/outdoor biting rates, biting times, blood-feeding preference, and resting locations of blood-fed mosquitoes) [15, 16]. Further, different locations within the same region can have entirely distinct vector communities. Therefore, it is important to evaluate mosquito sampling methods in various malaria-endemic regions with inter-regional vector diversity that may arise from differing climates, human activity patterns, seasonality, or other inherent ecological or entomological differences.

The gold standard for directly measuring human exposure and risk of malaria infection is the human landing collection (HLC), where a human collector sits with their legs exposed and captures mosquitoes seeking a blood meal. These collections potentially under-represent secondary vectors that only occasionally feed on humans, creating difficulty in assessing these species’ contributions to disease transmission. HLCs are also labour and cost intensive, and can be subject to local government and/or institutional review board (IRB) restrictions due to ethical concerns of collector safety [17], as well as being variable depending on the skill and mosquito attractiveness of the collectors. Several exposure-free traps have been developed to collect *Anopheles* mosquitoes attracted to humans [18, 19], including the Ifakara tent trap (ITT). Several iterations of the ITT have been tested in Tanzania and other countries in Africa and shown to be effective for collecting anthropophilic, indoor-biting malaria vectors such as *Anopheles gambiae* [16, 20–22]. The ITT was developed for collecting African malaria vectors, and had not been previously tested in Southeast Asia. In general, Indonesian anopheline mosquitoes do not favour entering ‘enclosed’ houses or traps (Laurent, Lobo, Malaria Transmission Consortium (MTC), unpublished). Few collection methods have been evaluated at any site in Indonesia, and many studies evaluating the distribution of malaria vectors are based on larval collections [23, 24].

This trap evaluation study was conducted in Indonesia, a geographically and ecologically varied archipelago with a large number of anopheline species. The country faces ongoing malaria transmission with an estimated 3.2–5.3 million cases in 2013 [2]. ITNs are presently estimated to be available to about 50% of the at-risk population, up from 20% at the time of this study in 2009 [2]. Distributions of anopheline species vary throughout the country, complicating transmission dynamics and potentially influencing the effectiveness of distributed ITNs.

The purpose of this study is to evaluate the best anopheline sampling methods in various transmission settings within Indonesia for continued mosquito collections, and to characterize resident primary and secondary vectors that may exhibit various bionomic traits (indoor/outdoor biting; anthropophagic/zoophagic, time of biting). Three villages were chosen as study sites to reflect different ecological and malaria transmission settings. Eight collection methods were tested in a Latin-square design to compare trap efficacy in each site. HLC (indoor and outdoor) was compared to two different sized human-occupied tents, the Ifakara tent trap C, CDC light traps, resting boxes (indoor and outdoor), resting pots (indoor and outdoor), and, in one site, a goat-baited tent trap. HLCs and tent trap collections were conducted hourly to study temporal patterns of activity. Since these traps may rely on different stimuli to capture mosquitoes and attractive cues may differ between species, the evaluation of their effectiveness was stratified by mosquito species.

## Methods

### Site description

**Fig. 1:**
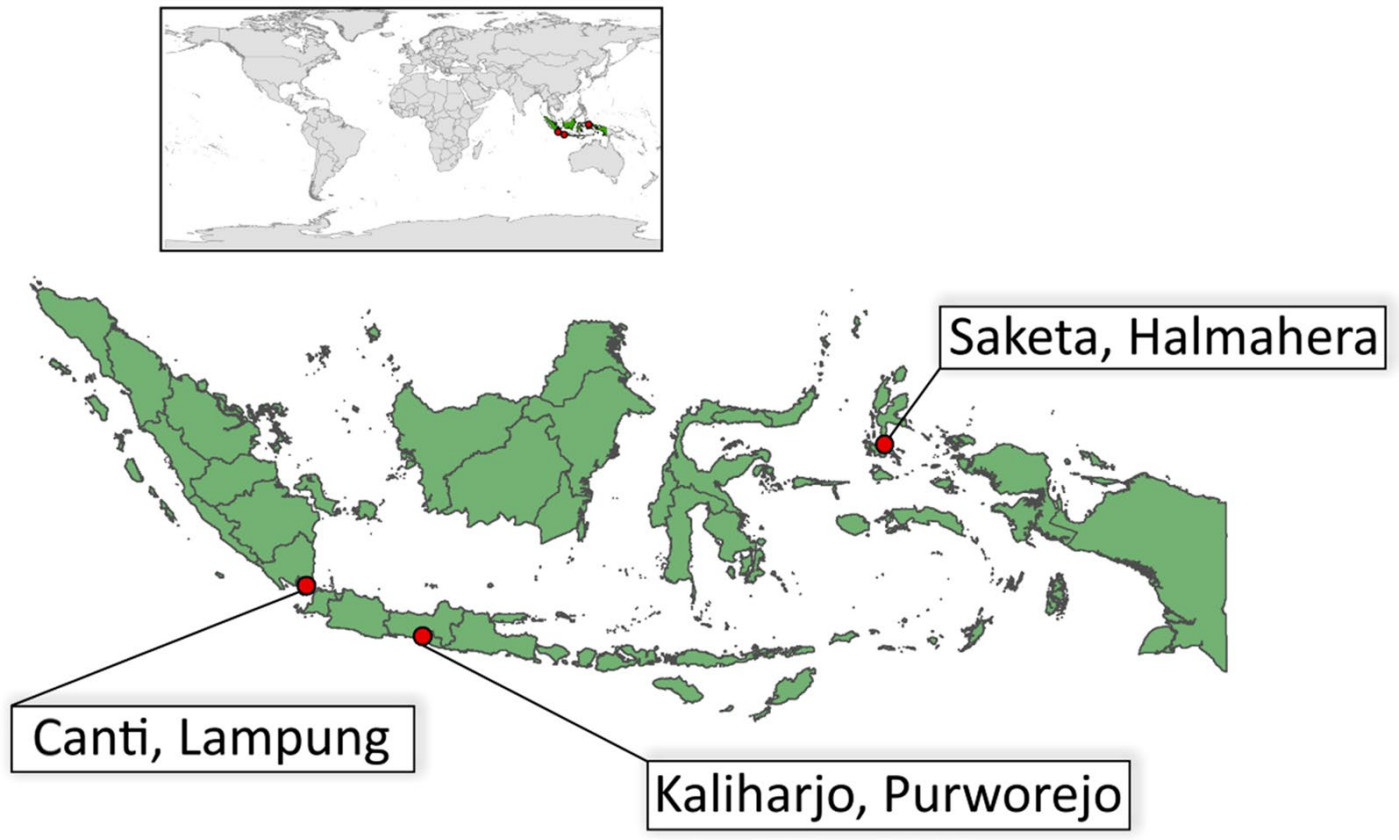
Locations of three collection sites across Indonesia. Sites of mosquito collection are labelled by village and province (referenced by village name in the text). Canti, Lampung; Kaliharjo, Purworejo; Saketa, Halmahera

These studies were carried out in three villages of varying transmission intensities across Indonesia (Fig. 1) during the period of peak mosquito densities, when possible. This period selected for trap evaluation were based on data from longitudinal collections over the preceding 2 years (Sukowati, pers. comm.).

#### Lampung—Canti village

This village is situated in western Indonesia, on the southern coast of Sumatra. This area is characterized by low to intermediate seasonal malaria endemicity. Houses representing local construction were randomly chosen, and were often adjacent to shrimp and fish farming facilities. Houses are typically made of brick or wood and plaster, with tiled roofs, and tend to have screens on some windows and eaves. Predominant activities in the area include fishing and shrimp farming that create slightly saline larval habitats which can be exploited by *Anopheles sundaicus*, the primary vector in this area [25]. This portion of the study was conducted over 16 nights in May–June, 2009.

#### Purworejo—Kaliharjo village

This village is situated in a highly forested and hilly area of central Java, with extremely low malaria incidence. Despite occasional epidemics, this area has been targeted for elimination of malaria. The primary land use surrounding the village is terraced rice farming. Houses are brick, wood, or thatch siding with tiled roofs. Structures have many holes and openings that allow mosquito entry and exit. *Anopheles aconitus* and *Anopheles balabacensis* are the most commonly collected local vectors using HLCs, with population peaks in February and July [26, 27]. *Anopheles aconitus* is a primary vector in other parts of central Java [27]. This portion of the study was conducted over 16 nights in July, 2009.

#### Halmahera—Saketa village

This village is located on the southwestern coast of Halmahera island in the North Maluku islands in eastern Indonesia. The region has the highest reported levels of malaria of the three study sites. However, longitudinal studies using HLCs have resulted in such low catches of *Anopheles* mosquitoes that trends in mosquito biting rates have been hard to determine (Sukowati, MTC, unpublished data). The area primarily consists of small fishing villages along the coast, with houses typically made of wood and plaster and metal rooftops. Many houses have open eaves, doors, and windows that allow mosquito entry and exit. This portion of the study took place over 8 nights in August, 2009, rather than the 16 nights in the other two sites, due to local political circumstances.

### Mosquito collection

Human landing collections took place from 18:00 to 06:00 each night. Trained collectors sat with their legs exposed and used a mouth aspirator and flashlight to collect landing mosquitoes. Indoor collectors sat inside a house, and outdoor collectors sat outside in a dark area. Mosquitoes were stored in a paper cup and collected every hour for processing. Collectors worked in 6-h shifts, switching collectors at midnight. Collectors were rotated indoors and outdoors to prevent bias associated with innate differences in attractiveness of individuals to mosquitoes.

The large tent traps were screen tents approximately 3 m × 5 m and 2 m high (Insta-Clip Six-sided Screen House, The Coleman Company, Inc.) placed outdoors. The tent is 6-sided with two doors on opposite sides, and is designed to be easily set up and taken down. A sleeping human was protected within a smaller, closed tent (‘Bug Hut’ 2-person tent, REI) inside the larger tent. In Saketa village, Halmahera, a goat-baited tent was also tested, wherein a goat was leashed loosely to a stake in the centre of the large tent. In both versions of the tent trap, mosquitoes resting on the inner walls of the large tent were collected using a mouth aspirator once per hour from 18:00 to 06:00.

The small tent traps were approximately 2 sq m (‘Bug Hut’ 2-person tent, REI) with a sleeping human protected within a smaller tent (Iguana BedNets 1-person pop-up tent, Iguana, LLC) inside. The tents were placed outdoors, and mosquitoes were collected hourly in the same manner as with the larger tent traps from 18:00 to 06:00.

The Ifakara tent trap C (ITT-C) has conical entry points on four sides that point inward, theoretically allowing mosquito entry to the upper part of the ITT-C but inhibiting exit. Inside the tent, a sleeping human on the ground level of the trap was protected from bites by a protective mesh barrier [16, 28]. The upper part of the trap was searched for mosquitoes at 06.00, to collect mosquitoes that may have entered from 18:00 to 06:00. The ITT-C was placed outside, but has recently been considered to represent an indoor-type environment [28].

Resting boxes were wooden 5-sided cubes, about 0.5 sq m per side and lined with black felt and open on one side. Resting pots were made locally from red clay, with fabric covering half the opening. To provide humidity, pots were moistened before use. These traps are intended to capture mosquitoes resting after blood meals, rather than those that are host seeking, and are constructed to create attractive resting spots. Two resting boxes and two resting pots were placed indoors, and two of each outdoors each night from 18:00 to 06:00. Resting boxes and pots were checked for resting mosquitoes in the morning, which were collected using mouth aspirators.

CDC light traps (CDC-LT) were placed indoors next to a human sleeping under an untreated bed net, which has been shown to increase trap efficiency by providing a CO_2_ and odourant source. The CDC-LT contains a battery-powered light above a fan that pulls mosquitoes through into a collection container. The protected human under the bed net serves as an attractive lure to bring host-seeking mosquitoes to the area for capture by the light trap. The light also serves as an attractant for some mosquito species. The light traps were set from 18:00 to 06:00 each night, and mosquitoes were collected in the morning.

To mitigate night and location effects, a 4 × 4 Latin square design was used for comparative evaluation of mosquito traps at each of the three sites (Table 1). These eight traps were evaluated in each of three sites previously described. Additionally, a goat-baited tent using the same tent as the large tent trap was tested in the high transmission site in Saketa village. Sampling sites were at least 100 m apart and tent traps were situated at least 10 m away from each other and the houses near where they were being tested to prevent trapping methods from impacting each other within the same household.

**Table 1:** Sampling schematic of a single round in one block

	House			
	1	2	3	4
Day 1	HLC indoors and outdoors	Resting pots and boxes indoors and outdoors	ITT-C outdoors and CDC-LT indoors	Small and large tent traps outdoors
Day 2	Small and large tent traps outdoors	HLC indoors and outdoors	Resting pots and boxes indoors and outdoors	ITT-C outdoors and CDC-LT indoors
Day 3	ITT-C outdoors and CDC-LT indoors	Small and large tent traps outdoors	HLC indoors and outdoors	Resting pots and boxes indoors and outdoors
Day 4	Resting pots and boxes indoors and outdoors	ITT-C outdoors and CDC-LT indoors	Small and large tent traps outdoors	HLC indoors and outdoors

A goat-baited tent was added to the sampling scheme with the resting pots and boxes only in Saketa, Halmahera

### Sample processing

Mosquitoes were collected using mouth aspirators from ITT, large and small tent traps, and HLC hourly from 18:00 to 06:00. Mosquitoes in resting pots, resting boxes, and CDC light traps were collected once in the morning between 06:00 and 06:30. Collections from each hour and for each trap type were held separately in paper cups until processing and morphological identification in the field [29].

Genomic DNA was isolated from individual specimens using a CTAB DNA extraction. A sequence of the ribosomal DNA internal transcribed spacer region two (rDNA ITS2) was used for molecular identification of mosquito species. This particular region, useful for differentiating *Anopheles* species complexes, is amplified using PCR with ITS2A and ITS2B primers [30]. This analysis was done on a small set of samples to confirm the presence of vector species. Not all of the collected samples were available to sequence for molecular species identity based on ITS2 sequence. The amplified fragments were visualized by electrophoresis on a 1% agarose gel. Prior to sequencing, fragments were purified using an enzyme cleanup: 2U of Exonuclease I (USB Corp, Cleveland, OH, USA), 1 U of Shrimp Alkaline Phosphatase (USB), and 1.8 μl of ddH20 were added to 8 μl of PCR product and incubated at 37 °C for 15 min followed by inactivation at 80 °C for 15 min. Purified products were sequenced directly using Sanger sequencing on an ABI 3730 xl DNA Analyzer Platform (Applied Biosystems). The ITS2 sequences were blasted against the NCBI GenBank nr database using BLASTn for molecular species identification.

The infection status of captured mosquitoes specimens was determined using the standard sandwich ELISA test for the detection of *Plasmodium falciparum*, *Plasmodium vivax*-210 and *Plasmodium vivax*-247 circumsporozoite (CS) proteins [31]. A subset of mosquitoes were analysed for *Plasmodium* infection using a multiplex PCR designed to detect *P. falciparum* and *P. vivax* [32].

### Statistical analysis

The mean catch differences between sampling methods were analysed independently for each site. The log(x + 1) transformation was used to achieve a normal distribution in the numbers of caught mosquitoes, and this transformed value was treated as the dependent variable and compared using ANOVA. The null hypothesis was there was no difference in nightly anopheline catch between sampling methods (no trap effect). A post hoc Tukey’s HSD test was performed to determine statistically significant differences between total catch due to trap and location effects in the experiment. These tests were performed using GraphPad Prism version 7.02 for Windows (GraphPad Software, La Jolla CA, USA).

Trap efficacy was evaluated in comparison to HLCs. Efficacy was estimated by dividing the mean trap catch by the mean HLC. Outdoor traps were compared to outdoor HLC and indoor traps were compared to indoor HLC. Relative catch numbers could only be compared for the trap evaluation in Canti due to the low catch rates in Kaliharjo and Saketa.

## Results

The number and species composition of *Anopheles* mosquitoes caught differed among the three study sites. In Canti village, Lampung, a total of 2353 anophelines were collected over 16 nights; all were morphologically identified as *An. sundaicus.* A sub-set (n = 68) of these mosquitoes were molecularly identified as *Anopheles epiroticus (An. sundaicus* species A) by ITS2 analysis (Table 2). In Kaliharjo village, Purworejo, 286 total anophelines were collected over 16 nights; they were morphologically identified as *An. aconitus* (n = 228), *An. balabacensis* (n = 46), *Anopheles barbirostris* (n = 11), and *Anopheles vagus* (n = 1) (Table 2). In Saketa village, Halmahera, 75 anophelines were collected over 8 nights; these were morphologically identified as *An. vagus* (n = 41), *Anopheles farauti* (n = 18), *Anopheles kochi* (n = 8), *Anopheles tessellatus* (n = 6), and *An. barbirostris* (n = 2) (Tables 2 and 3).

**Table 2:** *Anopheles* species collected by study site

	Canti, Lampung	Kaliharjo, Purworejo	Saketa, Halmahera	Total
*An. aconitus*		219		219
*An. balabancensis*		42		42
*An. barbirostris*		11	2	13
*An. farauti*			18	18
*An. kochi*			8	8
*An. sundaicus*	2350			2350
*An. tesselatus*			6	6
*An. vagus*		1	41	42
Total	2350	273	75	2695

Species were determined by morphological identification

**Table 3:** Collection method by species in three study sites

	HLC in	HLC out	Large tent	Small tent	ITT	CDC-LT	Resting box in	Resting box out	Resting jar in	Resting jar out	Total
*An. aconitus*	12	205	1				1				219
*An. balabancensis*	2	33	5	2							42
*An. barbirostris*		5	5	1	1					1	13
*An. farauti*		4				1				13	18
*An. kochi*			2							6	8
*An. sundaicus*	827	1277	104	82	13	41	2	2	3	2	2353
*An. tesselatus*	3	2	1								6
*An. vagus*		8								34	42
Total	844	1534	118	85	14	42	3	2	3	56	2701

Total numbers of eight anopheles species collected in each type of trap across all sites

**Fig. 2:**
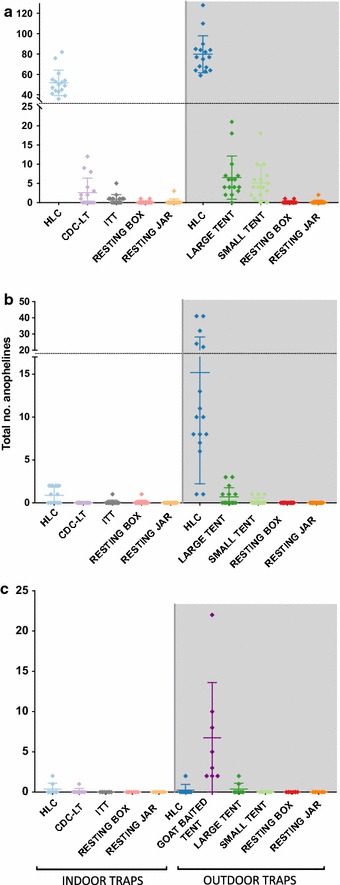
Total anophelines per trap over 16 catch nights. (Individual markers indicated collections from a single night in that trap at that location) in **a** Canti village, Lampung, indicating an anthropophilic population that prefers to feed outdoors but also feeds indoors. **b** Kaliharjo village, Purworejo, indicating an anthropophilic population that prefers to feed outdoors. **c** Saketa village, Halmahera, indicating a zoophilic population that prefers to feed outdoors. Traps are arranged by indoor (white background) and outdoor (grey background) traps. Note that a goat-baited tent was only evaluated in Saketa

There was a statistically significant trap effect on the mean daily catch of anophelines at each site, though overall trap effectiveness varied by site. The majority of specimens in Canti were collected by HLC, with 1277 and 827 in outdoor HLCs and indoor HLCs, respectively (Fig. 2a; Table 4). Large and small exposure-free tent traps collected 104 and 82 total anophelines, respectively, and the ITT-C collected 13. The indoor CDC light trap collected 41 anophelines; indoor and outdoor resting traps contributed very little to the overall collection numbers (Table 4). The collection method had a significant effect on the total number of captured anopheline mosquitoes (Table 4, ANOVA; F = 150.2, p < 0.0001) and a post hoc Tukey’s HSD test revealed that there was no significant difference between indoor/outdoor HLCs, though they were both significantly different from other traps (Additional file 1: Table S1, Tukey’s HSD; p < 0.0001). The large and small tent traps were not significantly different from each other but were significantly different from other traps (Additional file 1: Table S1, Tukey’s HSD; p < 0.0001). The CDC-LT was significantly different from the resting traps, but there were no significant differences between the ITT-C and the resting traps both in and outdoors. There was a significant location effect (ANOVA, p = 0.033), with one house significantly different than the other three tested.

**Table 4:** Total anophelines captured and mean catch with each collection method at three sites

	Canti, Lampung		Kaliharjo, Purworejo		Saketa, Halmahera	
	Total	Mean per night	Total	Mean per night	Total	Mean per night
Mean anopheline catch per trap by site						
HLC in	827	51.7	14	0.9	3	0.4
HLC out	1277	79.8	243	15.2	2	0.3
Large tent	104	0.2	11	0.0	3	0.4
Small tent	82	6.5	3	0.7	0	0.0
ITT	13	5.1	1	0.2	0	0.0
CDC-LT	41	0.8	0	0.1	1	0.0
Resting box in	2	2.6	1	0.0	0	0.1
Resting box out	2	0.1	0	0.1	0	0.0
Resting jar in	3	0.1	0	0.0	0	0.0
Resting jar out	2	0.1	0	0.0	0	0.0
Goat	–	–	–	–	54	6.8
Total	2353	147.1	273	17.1	63	7.9

Total catch was divided by the number of catch nights for each trap. There were 16 catch nights for Canti and Kaliharjo, and 8 for Saketa. Traps vary in efficacy by site, reflecting site-by-site variation in anopheline behaviour and densities

The efficiency of each type of trap in Canti was directly compared to catch rate of HLCs (Table 5). The CDC-LT and indoor resting pots and boxes were treated as indoor collections and compared with indoor HLCs, while all other traps were considered outdoor collections and compared with outdoor HLCs. All traps yielded lower numbers of *An. sundaicus* per night when compared to HLCs (Table 5). The large and small tent traps were the most effective alternatives, respectively yielding 8.1 and 6.4% compared to mean outdoor HLC. All other traps yielded less than 1% compared to HLC.

**Table 5:** Trap efficacy for *Anopheles sundaicus* in Canti village, Lampung

	Total *An. sundaicus* captured	Mean catch per night	Trap efficacy (compared to HLC)
Indoor traps			
HLC in	827	51.7	–
CDC-LT	41	2.6	0.05
Resting box in	2	0.1	0.00
Resting jar in	3	0.2	0.00
Outdoor traps			
HLC out	1277	79.8	–
Large tent	104	6.5	0.08
Small tent	82	5.1	0.06
ITT	13	0.8	0.01
Resting box out	2	0.1	0.00
Resting jar out	2	0.1	0.00

Trap efficacy was calculated by dividing the nightly mean of anophelines captured by each trap by the mean catch for human landing collection. CDC light trap, resting pot indoor, and resting box indoor collections were compared to HLC indoor catches. All other traps were compared to HLC outdoor catches. These collections occurred over 16 trap nights

Of 2347 *An. sundaicus* samples collected in Canti and screened by ELISA and sporozoite diagnostic PCR, 9 were found to be positive for *P. falciparum* and 62 were positive for *P. vivax* (Table 6). Of the *P. falciparum* positive specimens, 3 were captured with indoor HLC and 6 were captured with outdoor HLC. Of the *P. vivax* positive specimens, 16 were captured indoors with HLC, 31 were captured with outdoor HLC, 5 were captured in the indoor CDC-LT, 3 were captured in the large outdoor human-occupied tent, and 3 were captured in the small outdoor human-occupied tent (Table 6). In total, the sporozoite rates in Canti were 2.6% *P. vivax* and 0.4% *P. falciparum* (Table 6).

**Table 6:** Sporozoite-positive mosquitoes by collection method

	*An. sundaicus*	*An. aconitus*
HLC in	16 *P. v* 3 *P. f*	
HLC out	31 *P. v* 6 *P. f*	2 *P. v*
Large tent	3 *P. v*	
Small tent	3 *P. v*	
CDC-LT	5 *P. v*	
Total	62 *P. v* *9 P. f*	2 *P. v*
Sporozoite rate (%)	2.6% *P. v* *0.4% P. f*	0.7% *P. v*

*P. v* denotes *Plasmodium vivax* positives. *P. f* denotes *Plasmodium falciparum* positives. Sporozoite rates are calculated by dividing the number of positives by the total tested

*An. sundaicus* were collected in Canti, Lampung, *An. aconitus* were collected in Kaliharjo, Purworejo

In Kaliharjo, the majority of the 273 anophelines were captured in outdoor HLC, with 243 mosquitoes collected in these traps over 16 nights (Fig. 2b; Table 4). Indoor HLC yielded 14 mosquitoes; the large and small tent trap collected 11 and 3 anophelines, respectively. ITT-C and indoor resting boxes yielded one mosquito each over the collection period, and no mosquitoes were collected in the remaining traps. Outdoor HLC was significantly more effective than all other collection methods (Additional file 1: Table S1, Tukey’s HSD; p < 0.0001) and there was a significant trap effect (Table 4, ANOVA, p < 0.001). Indoor HLC and the large tent trap caught fewer anophelines and were statistically similar to each other, and all remaining traps were statistically similar (Additional file 1: Table S1). There was no trap location effect.

Of 220 samples analysed from Kaliharjo, 1 was PCR positive for *P. vivax,* and 1 was ELISA positive for *P. vivax* 247, a 0.7% overall *P. vivax* sporozoite positivity rate (Table 6). Both positive samples were captured with indoor HLC. These specimens were confirmed to be *An. aconitus* from ITS2 sequences.

In Saketa, a total of 63 anophelines were collected, with the majority collected in goat-baited large tent (Fig. 2c; Table 4). Fifty-four mosquitoes were captured in these traps over eight nights, compared to three mosquitoes captured in the human-occupied large tent over the same period. Indoor and outdoor HLCs yielded 3 and 2 anophelines, respectively. One mosquito was captured in the indoor CDC-LT. There was a significant trap effect for the goat-baited trap compared to other trapping methods (Table 4; Additional file 1: Table S1, Tukey’s HSD; p < 0.0001), as well as a significant location effect (Tukey’s HSD, p = 0.037) with one house location different from the means of the other three. Low sample numbers and variation between sites could be due to the shorter sampling period than at other sampling locations. No samples from Saketa were found to be *Plasmodium* positive.

**Fig. 3:**
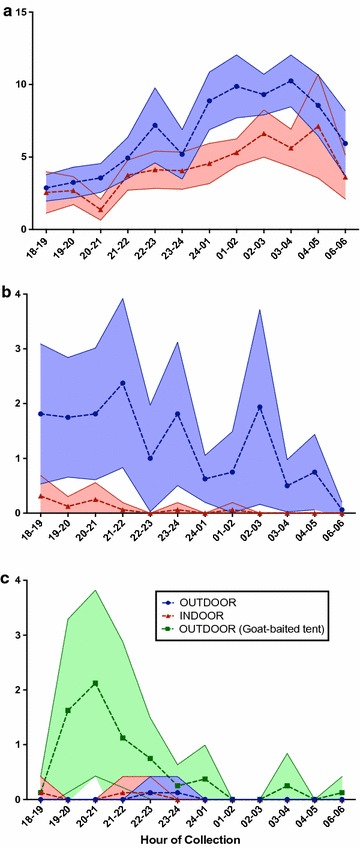
Comparisons of mean hourly anopheline catch rates by indoor and outdoor human landing collection over 16 nights. In **a** Canti village, Lampung: indicates biting is present throughout the night, but peaks in the pre-dawn early morning. **b** Kaliharjo village, Purworejo: indicates biting is present mostly outdoors and appears to decline by early morning. **c** Saketa village, Halmahera (8 nights of collection): indicates biting of humans is mostly absent throughout the night, but zoophilic behaviour is present in the early evening. A goat-baited tent was only evaluated in Saketa. Mean number of mosquitoes caught are plotted as points on an hourly basis, with 95% confidence interval bands

The biting profiles of mosquitoes by hour varied by species and by site (Fig. 3), with apparent distinct outdoor/indoor preference by site. In Canti, hourly counts of HLC showed mosquitoes were active throughout the night, but were more prevalent after midnight both indoors and outdoors (Fig. 3a). In Kaliharjo, mosquitoes were trapped relatively consistently throughout the sampling period outdoors, but were rarely collected indoors (Fig. 3b). In Saketa, low catch numbers prevent statistical interpretations. However, there is an apparent peak of anophelines early in the evening using goat-baited tent traps (Fig. 3c).

## Discussion

To evaluate trap efficacy at three sites in Indonesia representing different transmission environments, A Latin square design was used to compare seven collection techniques. This study suggests potential differences in mosquito behaviour in the three sites with respect to late night/early morning and indoor/outdoor biting behaviour. Trap efficacy likewise differs by site, and could be partly determined by species composition and location specific behavioural patterns (Table 4). In Canti, South Sumatra, indoor and outdoor HLCs were extremely effective for catching the anthropophilic primary vector of the area, *An. sundaicus*, including *P. falciparum*- and *P. vivax*-positive mosquitoes (Tables 2, 5, 6). In Kaliharjo, central Java, significantly more anophelines were collected in outdoor HLCs than any other trap tested, indicating that the local vector populations are exophagic but still anthropophilic. In Saketa, only a goat-baited tent collected meaningful numbers of anophelines, which may be highly zoophilic in this area, though this trap was not evaluated at the other two sites.

The comparative trap evaluation in Canti indicates that *An. sundaicus* are highly attracted to humans, both indoors and outdoors. While the host preference of these mosquitoes was not evaluated, they seek humans readily and are found infected with both *P. vivax* and *P. falciparum.* The elevated rate of *P. vivax* infection compared to *P. falciparum* is consistent with the risk estimates of malaria endemicity in Indonesia [33, 34]. While a number of mosquitoes were captured in three variations of tent traps in which humans are protected, the proportions of mosquitoes caught in these exposure-free tents were small compared to HLCs. Despite lower trap efficiency, the large and small human-occupied tents captured sporozoite positive mosquitoes, suggesting some possibility of their use as a monitoring tool in this area, though this may require using more traps to duplicate the capture rate of a single collector performing HLCs. However, the majority of *Plasmodium*-positive specimens were captured in HLCs, indicating that this remains a valid and useful primary method for vector monitoring here.

Anopheline collections in Canti consisted entirely of *An. sundaicus,* indicating that this species was the primary malaria vector in this village during the collection period, and also active both indoors and outdoors throughout the night with slightly elevated activity after midnight. *Anopheles sundaicus* is a saline-tolerant mosquito and the larvae may thrive in small fish and shrimp farming facilities, which are common in the area along the coast [25]. Management of these breeding sites may help to control populations of *An. sundaicus* and to reduce malaria transmission. Other work in the area has shown that there is a greater diversity of *Anopheles* species inland, but large populations of *An. sundaicus* may serve to sustain malaria transmission foci along the coast. The parasites circulating in these foci could influence transmission in nearby inland areas or wider ranges through human movement and travel. The indoor and outdoor HLCs of *An. sundaicus* in this study were comparable, indicating that this species is more willing to enter houses than vectors in other parts of Indonesia. This may make *An. sundaicus* more amenable to indoor control measures, such as ITNs or IRS.

In Kaliharjo, outdoor HLC was more effective than any other collection method, suggesting that anophelines in this area are attracted to humans, but are exophagic. If human-occupied tent traps are to be further evaluated in this area, they would likely need to be designed to have relatively unrestricted openings to encourage the local species to enter. Kaliharjo has a more diverse set of known vectors than Canti, and it is possible that this study missed some of the local anopheline species. *Anopheles aconitus, An. barbirostris* and *An. Balabacensis*, all captured in the area, are known secondary vectors. Further trap evaluations are necessary to fully explore the *Anopheles* species composition, and to make fully informed decisions on vector control monitoring strategies. Traps utilizing animal baits were not evaluated at all of the sites in this study, but have proven to be extremely successful in other areas of Indonesia and Southeast Asia and could help answer the question of local mosquito host preference [35].

Trapping in Saketa revealed that other trapping methods may be necessary for monitoring the diverse set of resident outdoor-biting and potentially zoophilic vectors. The goat-baited tent trap and other animal-baited traps should be further explored and expanded to other sampling locations in Indonesia. The malaria endemicity in this area is the highest of the three sampling locations in this study, yet the anopheline population was least attracted to humans in HLCs and in any type of human-occupied tent trap, yielding much lower mean nightly catches compared to the other two sites. A comparison of light traps and landing catches in Papua New Guinea (PNG) revealed that light traps are more effective than indoor or outdoor HLCs in catching two members of the *Anopheles punctulatus* complex [36]. Other studies have shown that this area contains a different set of vectors than PNG, with very few mosquitoes from the *An. punctulatus* complex present [37]. Extremely high catch rates have been shown using cow baits compared to humans in tents at this same collection site [38]. Due to low HLC catches in this and some other areas, meaningful biting rates are difficult to calculate and various epidemiological measures, such as entomological inoculation rate (EIR), may need to be calculated from non-entomological measures such as serological infection rates [39]. Further sampling would be required to accurately determine trap efficacy in this site.

## Conclusions

Human landing collection remains a valuable tool for estimating exposure of humans to potentially infectious bites from *Anopheles* mosquitoes, but may not be applicable in all transmission environments depending on resident *Anopheles* species behaviour. Indoor HLC failed to capture mosquitoes in two Indonesian sites, and HLCs were entirely ineffective in Saketa, a place with relatively higher malaria transmission than the other two study sites. In those areas where HLC is ineffective and malaria may be propagated by zoophilic or opportunistic mosquitoes, animal-baited trapping methods may be useful. Limitations of this study include small numbers of sampling nights, especially in Saketa, and the more cross-sectional nature of the study design. Longitudinal sampling efforts combined with monitoring malaria prevalence is necessary to better describe vector behaviour and species distributions during times of heightened or depressed transmission.

## Additional file

**Additional file 1: Table S1.** Tukey’s multiple comparisons tests for traps within each village.
