# Supplementary material for: Comparative evaluation of anopheline sampling methods in three localities in Indonesia
Source: Malar J. 2018 Jan 8;17:13. doi: 10.1186/s12936-017-2161-9 (PMC5759267; doi:10.1186/s12936-017-2161-9)
Supplement: Supplementary file 1 — Additional file 1: Table S1. Tukey’s multiple comparisons tests for traps within each village. [file 12936_2017_2161_MOESM1_ESM.docx]

**Table S1 Tukey's multiple comparisons tests for traps within each village**

Differences between the means of log-transformed nightly catch rates for each trap in each village tested. Differences between means are presented in bold, with 95% confidence intervals in square brackets. Differences are calculated relative to the trap listed in the row (a positive difference indicates the trap in the row had a higher mean catch rate than the trap in the column).

| **Canti Village** | **HLC in** | **HLC out** | **Large tent** | **Small tent** | **ITT** | **CDC-LT** | **Resting box in** | **Resting box out** | **Resting jar in** | **Resting jar out** |
| --- | --- | --- | --- | --- | --- | --- | --- | --- | --- | --- |
| **HLC in** |  | **-0.19** [-0.45 to 0.07] | **0.94** [0.68 to 1.20] | **1.04** [0.78 to 1.30] | **1.52** [1.26 to 1.78] | **1.36** [1.10 to 1.62] | **1.68** [1.41 to 1.94] | **1.68** [1.41 to 1.94] | **1.68** [1.42 to 1.94] | **1.67** [1.41 to 1.94] |
| **HLC out** |  |  | **1.13** [0.87 to 1.39] | **1.23** [0.97 to 1.50] | **1.71** [1.45 to 1.97] | **1.55** [1.29 to 1.81] | **1.86** [1.60 to 2.12] | **1.86** [1.60 to 2.12] | **1.87** [1.61 to 2.13] | **1.86** [1.60 to 2.12] |
| **Large tent** |  |  |  | **0.10** [-.02 to 0.37] | **0.58** [0.32 to 0.84] | **0.42** [0.16 to 0.68] | **0.73** [0.47 to 1.00] | **0.73** [0.47 to 1.00] | **0.74** [0.48 to 1.00] | **0.73** [0.47 to 1.00] |
| **Small tent** |  |  |  |  | **0.48** [0.22 to 0.74] | **0.32** [0.06 to 0.58] | **0.63** [0.37 to 0.89] | **0.63** [0.37 to 0.89] | **0.64** [0.37 to 0.89] | **0.63** [0.37 to 0.89] |
| **ITT** |  |  |  |  |  | **-0.16**  [-0.42 to -0.10] | **0.15**  [-0.11 to 0.41] | **0.15**  [-0.11 to 0.41] | **0.16**  [-0.10 to 0.42] | **0.15**  [-0.11 to 0.41] |
| **CDC-LT** |  |  |  |  |  |  | **0.31** [0.05 to 0.57] | **0.31** [0.05 to 0.57] | **0.32** [0.06 to 0.58] | **0.31** [0.05 to 0.57] |
| **Resting box in** |  |  |  |  |  |  |  | **0.00**  [-0.26 to 0.26] | **0.01**  [-0.27 to 0.25] | **0.00**  [-0.26 to 0.26] |
| **Resting box out** |  |  |  |  |  |  |  |  | **0.01**  [-0.25 to 0.27] | **0.00**  [-0.26 to 0.26] |
| **Resting jar in** |  |  |  |  |  |  |  |  |  | **0.01**  [-0.27 to 0.25] |
| **Resting jar out** |  |  |  |  |  |  |  |  |  |  |

P < 0.0001

P < 0.01

Not significant

| **Kaliharjo Village** | **HLC in** | **HLC out** | **Large tent** | **Small tent** | **ITT** | **CDC-LT** | **resting box in** | **resting box out** | **resting jar in** | **resting jar out** |
| --- | --- | --- | --- | --- | --- | --- | --- | --- | --- | --- |
| **HLC in** |  | **-0.85**  [-1.04 to -0.66] | **0.06**  [-0.14 to 0.25 | **0.16**  [-0.03 to 0.35] | **0.20** [0.01 to 0.39] | **0.22** [0.02 to 0.41] | **0.20** [0.01 to 0.39] | **0.22** [0.02 to 0.41] | **0.22** [0.02 to 0.41] | **0.22** [0.02 to 0.41] |
| **HLC out** |  |  | **0.90** [0.71 to 1.10] | **1.01** [0.82 to 1.20] | **1.05** [0.85 to 1.24] | **1.07** [0.87 to 1.26] | **1.05** [0.85 to 1.24 | **1.07** [0.87 to 1.26] | **1.07** [0.87 to 1.26] | **1.07** [0.87 to 1.26] |
| **Large tent** |  |  |  | **0.11**  [-0.09 0.30] | **0.14**  [-0.05 to 0.34] | **0.16**  [-0.03 to 0.35] | **0.14**  [-0.05 to 0.34] | **0.16**  [-0.03 to 0.35] | **0.16**  [-0.03 to 0.35] | **0.16**  [-0.03 to 0.35] |
| **Small tent** |  |  |  |  | **0.04**  [-0.15 to 0.23] | **0.06**  [-0.14 to 0.25] | **0.04**  [-0.15 to 0.23] | **0.06**  [-0.14 to 0.25] | **0.06**  [-0.14 to 0.25] | **0.06**  [-0.14 to 0.25] |
| **ITT** |  |  |  |  |  | **0.02**  [-0.17 to 0.21] | **0.00**  [-0.19 to 0.19] | **0.02**  [-0.17 to 0.21] | **0.02**  [-0.17 to 0.21] | **0.02**  [-0.17 to 0.21] |
| **CDC-LT** |  |  |  |  |  |  | **-0.02**  [-0.21 to 0.17] | **0.00**  [-0.19 to 0.19] | **0.00**  **[-0.19 to 0.19]** | **0.00**  **[-0.19 to 0.19]** |
| **Resting box in** |  |  |  |  |  |  |  | **0.02**  [-0.17 to 0.21] | **0.02**  [-0.17 to 0.21] | **0.02**  [-0.17 to 0.21] |
| **Resting box out** |  |  |  |  |  |  |  |  | **0.00**  **[-0.19 to 0.19]** | **0.00**  **[-0.19 to 0.19]** |
| **Resting jar in** |  |  |  |  |  |  |  |  |  | **0.00**  **[-0.19 to 0.19]** |
| **Resting jar out** |  |  |  |  |  |  |  |  |  |  |

P < 0.0001

P < 0.05

Not significant

| **Saketa Village** | **Goat-baited tent** | **HLC in** | **HLC out** | **Large tent** | **Small tent** | **ITT** | **CDC-LT** | **resting box in** | **resting box out** | **resting jar in** | **resting jar out** |
| --- | --- | --- | --- | --- | --- | --- | --- | --- | --- | --- | --- |
| **Goat-baited tent** |  | **0.67** [0.44 to 0.91] | **0.71** [0.48 to 0.94] | **0.67** [0.44 to 0.91] | **0.77** [0.54 to 1.00] | **0.77** [0.54 to 1.00] | **0.73** [0.50 to 0.97] | **0.77** [0.54 to 1.00] | **0.77** [0.54 to 1.00] | **0.77** [0.54 to 1.00] | **0.77** [0.54 to 1.00] |
| **HLC in** |  |  | **0.04**  [-0.19 to 0.27] | **0.00**  [-0.23 to 0.23] | **0.10**  [-0.13 to 0.33] | **0.10**  [-0.13 to 0.33] | **0.06**  [-0.17 to 0.29] | **0.10**  [-0.13 to 0.33] | **0.10**  [-0.13 to 0.33] | **0.10**  [-0.13 to 0.33] | **0.10**  [-0.13 to 0.33] |
| **HLC out** |  |  |  | **-0.04**  [-0.27 to 0.19] | **0.06**  [-0.17 to 0.29] | **0.06**  [-0.17 to 0.29] | **0.02**  [-0.21 to 0.25] | **0.06**  [-0.17 to 0.29] | **0.06**  [-0.17 to 0.29] | **0.06**  [-0.17 to 0.29] | **0.06**  [-0.17 to 0.29] |
| **Large tent** |  |  |  |  | **0.10**  [-0.13 to 0.33] | **0.10**  [-0.13 to 0.33] | **0.06**  [-0.29 to 0.17] | **0.10**  [-0.13 to 0.33] | **0.10**  [-0.13 to 0.33] | **0.10**  [-0.13 to 0.33] | **0.10**  [-0.13 to 0.33] |
| **Small tent** |  |  |  |  |  | **0.00**  [-0.23 to 0.23] | **-0.04**  [-0.27 to 0.19] | **0.00**  [-0.23 to 0.23] | **0.00**  [-0.23 to 0.23] | **0.00**  [-0.23 to 0.23] | **0.00**  [-0.23 to 0.23] |
| **ITT** |  |  |  |  |  |  | **-0.04**  [-0.27 to 0.19] | **0.00**  [-0.23 to 0.23] | **0.00**  [-0.23 to 0.23] | **0.00**  [-0.23 to 0.23] | **0.00**  [-0.23 to 0.23] |
| **CDC-LT** |  |  |  |  |  |  |  | **0.04**  [-0.19 to 0.27] | **0.04**  [-0.19 to 0.27] | **0.04**  [-0.19 to 0.27] | **0.04**  [-0.19 to 0.27] |
| **Resting box in** |  |  |  |  |  |  |  |  | **0.00**  [-0.23 to 0.23] | **0.00**  [-0.23 to 0.23] | **0.00**  [-0.23 to 0.23] |
| **Resting box out** |  |  |  |  |  |  |  |  |  | **0.00**  [-0.23 to 0.23] | **0.00**  [-0.23 to 0.23] |
| **Resting jar in** |  |  |  |  |  |  |  |  |  |  | **0.00**  [-0.23 to 0.23] |
| **Resting jar out** |  |  |  |  |  |  |  |  |  |  |  |

P < 0.0001

P < 0.05
